# Supplementary material for: MetaRibo-Seq measures translation in microbiomes
Source: Nat Commun. 2020 Jun 29;11:3268. doi: 10.1038/s41467-020-17081-z (PMC7324362; doi:10.1038/s41467-020-17081-z)
Supplement: Supplementary file 10 — Supplementary Data 7 [file 41467_2020_17081_MOESM10_ESM.zip › File2/Confidence_High_Taxonomy/146009_out.krona.html]

Javascript must be enabled to view this page.

members
magnitude
magnitudeUnassigned
count
unassigned
taxon
rank

146009\_out

2

2
superkingdom
2

phylum
2
1239

2
class
186801

2
order
186802

541000
1
family

species
1

SRS1041036\_contig\_number\_432
1952281

1
species

SRS078242\_contig\_number\_contig-100\_5830.5830
1950886
